# Organ transplantation and gender differences: a paradigmatic example of intertwining between biological and sociocultural determinants

**DOI:** 10.1186/s13293-016-0088-4

**Published:** 2016-07-28

**Authors:** Francesca Puoti, Andrea Ricci, Alessandro Nanni-Costa, Walter Ricciardi, Walter Malorni, Elena Ortona

**Affiliations:** 1 National Transplant Center (CNT), Istituto Superiore di Sanità, Rome, Italy; 2 Section of Gender Medicine, Istituto Superiore di Sanità, Rome, Italy; 3 Department of Therapeutic Research and Medicine Evaluation, Section of Cell Aging and Degeneration, Istituto Superiore di Sanità, viale Regina Elena 299, 00161, Rome, Italy

**Keywords:** Transplantation, Sex differences, Gender differences

## Abstract

Organ transplantation, e.g., of the heart, liver, or kidney, is nowadays a routine strategy to counteract several lethal human pathologies. From literature data and from data obtained in Italy, a striking scenario appears well evident: women are more often donors than recipients. On the other hand, recipients of organs are mainly males, probably reflecting a gender bias in the incidence of transplant-related pathologies. The impact of sex mismatch on transplant outcome remains debated, even though donor-recipient sex mismatch, due to biological matters, appears undesirable in female recipients. In our opinion, the analysis of how sex and gender can interact and affect grafting success could represent a mandatory task for the management of organ transplantation.

## Background

Thanks to the improvement of surgical techniques and immunosuppressant treatments, organ transplantation is nowadays considered as a routine strategy for patients suffering a series of pathological states of a number of organs such as the heart, kidney, or liver. Transplant of these organs in patients with end-stage diseases has been demonstrated to significantly improve survival and/or quality of life. However, a refinement of therapeutic strategies, including immunosuppressant treatments, as well of the comprehension of the pathogenetic mechanisms leading to successful or ineffective transplantation outcome appears mandatory. Several lines of evidence suggested that successful organ transplantation could depend upon a plethora of factors. Among these are race, size, age, weight, and even sex/gender [1]. Gender-related factors, i.e., sociocultural matters, or sex-related factors, i.e., biological determinants, appeared in fact as a pivotal matter in this scenario, being capable of influencing transplant outcome. In this *Commentary*, we would like to briefly underscore some of these critical points in order to stimulate a reappraisal of the gender/sex issue in transplantation studies and practice. We underline the strict intertwining between sociocultural and biological questions as pivotal issues in this field.

### Impact of gender on organ transplantation

The gender of donors and recipients is involved in the entire process, including organ donation and transplant surgery. In general, women seem to have more self-sacrifice and sense of responsibility than men [2]. As a consequence, it has been observed that women are more predisposed to donate their organs. In fact, in cost-free living donation, two thirds of all organs were donated by women [3]. In contrast, women are less disposed than males to accept transplant surgery [2]. Despite comprising 35 % of transplants, the number of female transplant recipients continued to decline. Several factors have been suggested to explain these differences [1]. Nowadays, women and men present different social, economic, and cultural roles, and a disparity of knowledge may exist. In fact, women were considered to have less information about transplantation diagnosis and therapy. However, besides these psychosocial aspects, another important factor should be considered to explain the above reported gender bias: men have a higher incidence of end-stage diseases that necessitate a transplant and are more inclined to hypertension or ischemic heart disease, leading to their inappropriateness as donors.

Regarding graft outcome, male recipients have been observed to have a worse prognosis than females and this could be partially explained by the observation that women have better immunosuppressant compliance than men; they undergo follow-up visits and habit change and show more concern with regard to protecting graft function [4].

### Impact of sex on organ transplantation

Several clinical studies have connected the use of female donor organs as a risk factor for death and rejection [5]. In renal transplantation, female donor kidneys have a worse 5-year survival [6, 7] and this observation could be explained by the lower number of nephrons in the female kidney in comparison to men [8]. In addition, animal experiments suggested that kidneys of females express more HLA antigens and are more antigenic [5]. Moreover, male grafts are less susceptible to nephrotoxic effects of some immunosuppressants than female grafts [7]. Long-term retrospective studies in renal transplants revealed that male recipients undergo a worse survival in comparison to females [9]. It can be hypothesized that protection afforded by hormones in women could result in their better long-term prognosis. Estradiol can in fact improve graft function, preserve graft architecture, and diminish cellular infiltration, including mononuclear cell infiltration [10].

The impact of sex mismatch on transplant outcome still remains a matter of debate. Several studies reported that female donor to male recipient grafts seems to have a worst prognosis in particular for liver [11–13] and heart transplantation [14]. In particular, in a recent single-center retrospective study, Schoening et al. [15], evaluating the effect of sex differences on long-term graft survival after liver transplant, found that female donor-male recipient combination showed the worst graft survival. They suggested that this event could be caused by the reduced female donor “quality” (female donors were significantly older, died significantly more frequently from cerebrovascular causes and less frequent by trauma) and by unfavorable characteristics of male recipients (higher incidence of hepatocellular carcinoma in the male recipient group). Interestingly, in studies carried out in animal models, livers from female rats have been demonstrated to present an increased acidosis during transplant-associated ischemia in comparison with livers from male rats; this sex difference in the liver’s metabolic response to ischemia appeared estrogen-mediated and could have a significant influence on the outcome of transplantation [16, 17]. Since a similar sex-dependent metabolic response has been found also in myocardial function [18], the possibility that this sexual disparity could influence cardiac transplants cannot be ruled out. In contrast with these studies, other studies on renal transplantation observed that male donor to female recipient combination is an independent risk factor for poor graft survival [19, 20] and the significantly higher percentage of H-Y antibody production in the male donor-female recipient population could play a role in this phenomenon [21]. Regarding heart transplant patients, the observation that donor-recipient sex mismatch could result in a lower survival suggested that sex mismatch can be undesirable in female, as well as male, recipients [22].

### Discussion

An important point to be considered in the evaluation of the possible sex/gender disparity in transplantation policy is the limited availability of data worldwide. Legislation differs in fact significantly among western countries, some of which lack specific rules. In Italy, organ donation and transplantation activities are coordinated by law by the National Transplant Centre (CNT) which, in collaboration with 90 transplantation centers operating in Italy, should ensure the quality and traceability of the entire process all over the national territory. To this purpose, all donations, patients, and transplants performed in Italy are recorded on the Transplant Information System (SIT). Transplant activity data registered in SIT since 2002 (the last 13 years) are reported in Table 1 in which the gender of donors and recipients in transplants from living and cadaveric donors are shown. In line with those reported above, i.e., a better capacity to donate of the female gender in comparison with the male gender, we observed that 66 % of living donors were women (in Italy, all living donors are unpaid), whereas 65 % of total transplants were performed in males. The main diseases leading to transplantation in our patients were the following: (i) for kidney transplants, chronic glomerulonephritis, and Berger disease (67 and 80 % in males, respectively); (ii) for liver transplants, hepatitis C virus cirrhosis, alcoholic cirrhosis, and hepatocellular carcinoma (77, 86, and 85 % in males, respectively); and (iii) for heart transplants, idiopathic dilated cardiomyopathy (78 % in males). Interestingly, these percentages were comparable with the gender differences in the distribution of the same diseases in the general population. Therefore, in our opinion, the gender bias in access to transplantation, i.e., the fact that recipients of organs are mainly males, could reflect the gender bias in the incidence of transplant-related pathologies.

**Table 1:** Transplant activity in Italy 2002–2015

Living donor transplants						
	Donor					
Recipient	Female		Male		Total	
Female	507	18 %	498	17 %	1005	**35 %**
Male	1379	48 %	467	16 %	1846	**65 %**
Total	1886	**66 %**	965	**34 %**	2851	100 %
Cadaveric donor transplants						
	Donor					
Recipient	Female		Male		Total	
Female	6636	16 %	6102	15 %	12,738	**31 %**
Male	11,477	28 %	16,254	40 %	27,731	**69 %**
Total	18,113	**45 %**	22,356	**55 %**	40,469	100 %
Heart TX						
	Donor					
Recipient	Female		Male		Total	
Female	616	16 %	309	8 %	925	**23 %**
Male	853	22 %	2188	55 %	3041	**77 %**
Total	1469	**37 %**	2497	**63 %**	3966	100 %
Liver TX						
	Donor					
Recipient	Female		Male		Total	
Female	2039	15 %	1532	11 %	3571	**26 %**
Male	4065	30 %	5956	44 %	10,021	**74 %**
Total	6104	**45 %**	7488	**55 %**	13,592	100 %
Kidney TX						
	Donor					
Recipient	Female		Male		Total	
Female	3603	17 %	4105	19 %	7708	**36 %**
Male	6271	29 %	7613	35 %	13,884	**64 %**
Total	9874	**46 %**	11,718	**54 %**	21,592	100 %

Note that in living donor transplants, females are two thirds (65 %) of donors and only one thirds (35 %) of recipients. In cadaveric donor transplants, the percentage of female recipients is similar to the previous (31 %) while female donors are less than half (45 %) of total cadaveric donors. The percentages of female and male donors and recipients are represented in bold

*TX* transplant

**Fig. 1:**
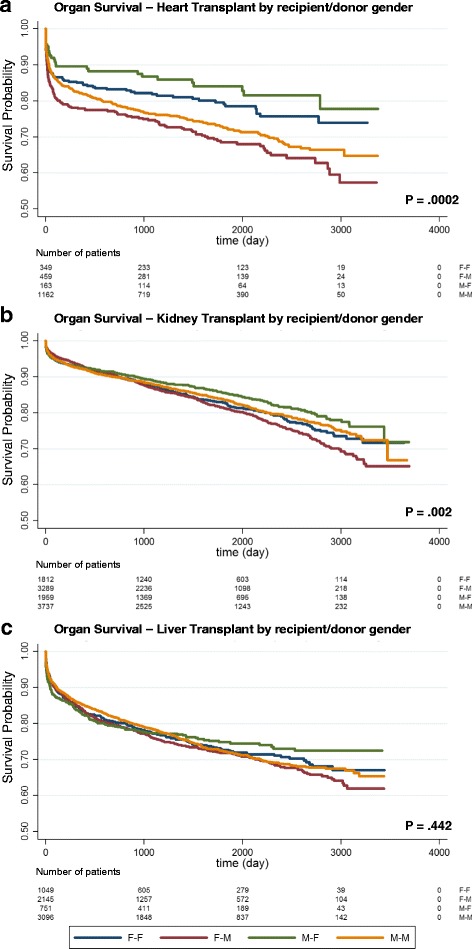
**a**–**c** Graft survival in Italy. Kaplan-Meier estimates of graft survival of all the transplants performed in the period 2006–2013, excluding re-transplants, combined transplants, and transplants with more than one risk factor (according to risk assessment protocols adopted in Italy since the year 2006). The log-rank test is used to test the null hypothesis. Latest update of graft follow-up: year 2016

Evaluating the graft survival by Kaplan-Meier analyses (Fig. 1), we observed that the donor female (F)–recipient male (M) mismatch presented (i) a significant decrease of graft survival after heart transplantation (Fig. 1a, *P* = .0002), (ii) a significant decrease of graft survival in the long run after kidney transplantation (Fig. 1b, *P* = .002), and (iii) a not significant trend of decrease of graft survival after liver transplantation (Fig. 1c, *P* = .442). On the other hand, the donor M–recipient F mismatch showed the best long-term survival, in particular for heart transplantation. However, the number of variables to be considered before identifying proper gender differences appears to be quite complex and a lot of data regarding, among others, the severity of the disease in recipients and the quality of transplanted organs should be taken into account. Furthermore, the mean age (± standard deviation) of donors appears higher for females than that for males (F: 53 ± 18.1; M: 46 ± 19.6), whereas the mean age of recipients is higher in males than that in females (F: 47 ± 16; M: 50 ± 14.1) so that we can hypothesize that the age of both the donor and the recipient can represent a critical risk factor exerting a significant influence on the graft survival.

A multivariate analysis could represent in our mind, the unique and proper statistical approach capable of providing valuable information about possible gender disparity in organ transplantation allowing to understand the strict intertwining between biological and sociocultural determinants.

## Conclusion

The impact of sex mismatch on transplant outcome still remains a matter of debate. Both gender- and sex-related aspects might affect the donation, the access, and the outcome of transplantation. In particular, how sex and gender interact and affect graft success should be taken into account in the management of organ-transplanted patients. In our opinion, this appears as a mandatory task to be promoted, developed, and regulated.

## Abbreviations

CNT, National Transplant Centre; F, female; M, male; SIT, Transplant Information System

## References

[1] Ge F, Huang T, Yuan S, Zhou Y, Gong W (2013). Gender issues in solid organ donation and transplantation. Ann Transplant.

[2] Legato MJ, Sanfey IH, Frcsi F, Frcsi (2004). Gender-specific issues in organ transplantation. Principles of gender-specific medicine.

[3] Steinman JL (2006). Gender disparity in organ donation. Gend Med.

[4] Rosenberger J, Geckova AM, van Dijk JP, Nagyova I, Roland R, van den Heuvel WJ (2005). Prevalence and characteristics of noncompliant behaviour and its risk factors in kidney transplant recipients. Transpl Int.

[5] Zeier M, Döhler B, Opelz G, Ritz E (2002). The effect of donor gender on graft survival. J Am Soc Nephrol.

[6] Głyda M, Czapiewski W, Karczewski M, Pięta R, Oko A (2011). Influence of donor and recipient gender as well as selected factors on the five-year survival of kidney graft. Pol Przegl Chir.

[7] Shibue T, Kondo K, Iwaki Y, Terasaki PI. Effect of sex on kidney transplants. Clin Transplant. 1987;351–60. 3154434

[8] Kasiske BL, Umen JA (1986). The influence of age, sex, race and body habitus on kidney weight in humans. Arch Pathol Lab Med.

[9] Chen PD, Tsai MK, Lee CY, Yang CY, Hu RH, Lee PH, Lai HS (2013). Gender differences in renal transplant graft survival. J Formos Med Assoc.

[10] Muller V, Szabo A, Viklicky O, Pörtl S, Philipp T (1999). Sex hormones and gender-related differences: their influence on chronic renal allograft rejection. Kidney Int.

[11] Yoshizumi T, Shirabe K, Taketomi A, Uchiyama H, Harada N, Ijichi H (2012). Risk factors that increase mortality after living donor liver transplantation. Transplantation.

[12] Brooks BK, Levy MF, Jennings LW, Abbasoglu O, Vodapally M, Goldstein RM (1996). Influence of donor and recipient gender on the outcome of liver transplantation. Transplantation.

[13] Rustgi VK, Marino G, Halpern MT (2002). Role of gender and race mismatch and graft failure in patients undergoing liver transplantation. Liver Transplant.

[14] Kaczmarek I, Meiser B, Beiras-Fernandez A, Guethoff S, Überfuhr P, Angele M (2013). Gender does matter: gender-specific outcome analysis of 67,855 heart transplants. Thorac Cardiovasc Surg.

[15] Schoening WN, Helbig M, Buescher N, Andreou A, Bahra M, Schmitz V (2016). Gender matches in liver transplant allocation: matched and mismatched male-female donor-recipient combinations; long-term follow-up of more than 2000 patients at a single center. Exp Clin Transplant.

[16] Wittnich C, Belanger MP, Askin N, Boscarino C, Wallen WJ (2004). Lower liver transplant success in females: gender differences in metabolic response to global ischemia. Transplant Proc.

[17] Soric S, Belanger MP, Askin N, Wittnich C (2007). Impact of female sex hormones on liver tissue lactic acidosis during ischemia. Transplantation.

[18] Wittnich C, Tan L, Wallen J, Belanger M (2013). Sex differences in myocardial metabolism and cardiac function: an emerging concept. Pflugers Arch.

[19] Zukowski M, Kotfis K, Biernawska J, Zegan-Barańska M, Kaczmarczyk M, Ciechanowicz A (2011). Donor-recipient gender mismatch affects early graft loss after kidney transplantation. Transplant Proc.

[20] McGee J, Magnus JH, Islam TM, Jaffe BM, Zhang R, Florman SS, et al. Donor-recipient gender and size mismatch affects graft success after kidney transplantation. J Am Coll Surg. 2010;210:718-725.e1, 725-6. 10.1016/j.jamcollsurg.2009.12.032 PMC2887601 20421037

[21] Scott DM, Ehrmann IE, Ellis PS, Chandler PR, Simpson E (1997). Why do some females reject males? The molecular basis for male-specific graft rejection. J Mol Med.

[22] Kittleson MM, Shemin R, Patel JK, Ardehali A, Kawano M, Davis S (2011). Donor-recipient sex mismatch portends poor 10-year outcomes in a single-center experience. J Heart Lung Transplant.

